# Liquid Biopsy for Cancer Cachexia: Focus on Muscle-Derived microRNAs

**DOI:** 10.3390/ijms22169007

**Published:** 2021-08-20

**Authors:** Roberta Belli, Elisabetta Ferraro, Alessio Molfino, Raffaella Carletti, Federica Tambaro, Paola Costelli, Maurizio Muscaritoli

**Affiliations:** 1 Department of Translational and Precision Medicine, Sapienza University, 00185 Rome, Italy; alessio.molfino@uniroma1.it (A.M.); raffaella.carletti@uniroma1.it (R.C.); federica.tambaro@uniroma1.it (F.T.); 2 Unit of Cell and Developmental Biology, Department of Biology, University of Pisa, 56126 Pisa, Italy; elisabetta.ferraro@unipi.it; 3 Department of Clinical and Biological Sciences, University of Torino, 10124 Torino, Italy; paola.costelli@unito.it

**Keywords:** cancer cachexia, liquid biopsy, miRNAs, biomarkers

## Abstract

Cancer cachexia displays a complex nature in which systemic inflammation, impaired energy metabolism, loss of muscle and adipose tissues result in unintentional body weight loss. Cachectic patients have a poor prognosis and the presence of cachexia reduces the tolerability of chemo/radio-therapy treatments and it is frequently the primary cause of death in advanced cancer patients. Early detection of this condition could make treatments more effective. However, early diagnostic biomarkers of cachexia are currently lacking. In recent years, although solid biopsy still remains the “gold standard” for diagnosis of cancer, liquid biopsy is gaining increasing interest as a source of easily accessible potential biomarkers. Moreover, the growing interest in circulating microRNAs (miRNAs), has made these molecules attractive for the diagnosis of several diseases, including cancer. Some muscle-derived circulating miRNA might play a pivotal role in the onset/progression of cancer cachexia. This topic is of great interest since circulating miRNAs might be easily detectable by means of liquid biopsies and might allow an early diagnosis of this syndrome. We here summarize the current knowledge on circulating muscular miRNAs involved in muscle atrophy, since they might represent easily accessible and promising biomarkers of cachexia.

## 1. Introduction

Cachexia is a multifactorial disorder associated with chronic diseases, such as cancer, chronic heart failure, chronic obstructive pulmonary disease, and chronic kidney disease [1].

Cachexia in cancer affects about half of the patients and is the direct cause of death in about 20% of them [2,3,4,5,6,7]. Cancer cachexia (CC) is a life-threatening disorder driven by a complex interaction between tumor-and host-related factors [8,9,10] and its underlying mechanisms are not completely clarified [11]. A major hallmark of cachexia is the involuntary weight loss due to low muscle mass, with or without loss of fat mass [12]. The pathophysiology of muscle wasting during cachexia is characterized by a negative protein and energy balance, caused by a variable combination of reduced food intake and metabolic abnormalities [13,14,15]. Chronic inflammation seems to be one of the major drivers of muscle wasting in cancer cachexia [16,17,18]. Also, altered glucose metabolism and insulin resistance are associated with cancer cachexia and their contribution to the progression of skeletal muscle wasting has been reviewed by Masi and collaborators [16,17,18]. In spite of its detrimental influence on patients’ quality of life [8,9,10], morbidity and mortality, and the wide circulation of the diagnostic criteria [17], CC is still largely unrecognized, underdiagnosed and undertreated [19]. Currently no biomarkers of cancer cachexia are present [20]. In the last ten years, several molecules have been proposed as biomarkers of cachexia including pro-inflammatory cytokines (e.g., interleukine 6, IL-6 and tumor necrosis factor alpha, TNF-α), hormones (e.g., leptin and ghrelin) and peptides such as C-terminal agrin fragment (CAF) [21,22,23]. However, none of these are specific to this syndrome and none of these fully satisfy the characteristics of a good biomarker in CC [21,22,23].

It has been suggested that some cellular mediators, including specific microRNAs (miRNAs), released by host tissues or by tumor cells, might contribute to the development of cachexia [23,24,25]. Detection of muscle-derived miRNAs in the blood might allow for the recognition of muscle derangements occurring in cachexia from the early stage.

In this review, we report on the most recent evidence on the modulation of circulating muscle-derived miRNAs during cancer cachexia, both in animal models and humans. We consider muscle-derived miRNAs those expressed- exclusively or not- by skeletal muscles. We hypothesize that, in the near future, the circulating forms of some of these miRNAs could be exploited as biomarkers of cancer cachexia in liquid biopsies.

## 2. Liquid Biopsy

### 2.1. Liquid Biopsy in Cancer

Liquid biopsy is a technique that is gaining increasing interest, especially in the field of oncology. Although solid biopsy still remains the “gold standard” for diagnosis and treatment choice for many types of cancer [26], liquid biopsy utilizes body fluids as surrogate tissues to provide information on cancer.

Originally, the liquid biopsy in the field of cancer was used to study mainly the circulating tumor cells (CTCs). Indeed, the U.S. National Cancer Institute (NCI) defined liquid biopsy as “a test done on a sample of blood to look for cancer cells from a tumor that are circulating in the blood or for pieces of DNA from tumor cells that are in the blood” [27]. However, in the last few years, besides CTCs, numerous circulating elements have been identified in liquid biopsies [28], such as cell-free circulating nucleic acids (cfNAs), microvesicles, and exosomes [29]. More schematically, in a liquid biopsy, we can non-invasively detect circulating cellular (i.e., CTCs), subcellular (extracellular vesicles) and molecular (e.g., DNA, miRNA, lncRNA, mRNA, proteins) [30,31] elements deriving from a tumor, which might allow the detection of tumor-specific genetic aberration, or RNA or protein alterations [30].

The detection and isolation of these circulating particles as sources of cancer genomic and proteomic information is commonly performed using blood samples [29]. However, CTCs, cfNAs and extracellular vesicles are also present in other biological fluids such as urine, saliva and cerebrospinal-fluid [32,33,34,35,36,37,38].

The scientific community’s growing interest in liquid biopsy depends on the possibility to obtain relevant information on the tumor and to learn about its molecular dynamic changes with minimally invasive methods, less dangerous and less expensive compared to surgical biopsies [39,40,41,42,43,44,45]. Indeed, although giving direct information, tissue biopsies have some disadvantages such as lack of indications regarding the spatial and temporal heterogeneity of the tumor [46], limited accessibility to the tumor tissue which could increase the possibility of false negative results [46], and bleeding or infections as a consequence of an invasive biopsy or excisional procedure [47]. Vice versa, a liquid biopsy obtained with a routine blood draw overcomes many of these limitations [48]; liquid biopsy could be used as a rapid tool for the diagnosis and monitoring of tumor progression and, possibly, for prediction of treatment response, detection of recurrence, and traceability of tumor genome evolution over time [42,43,49,50], which would be almost impossible with solid biopsy (Table 1).

Although the potential of liquid biopsy is emerging as a diagnostic and prognostic tool, some issues need to be solved before liquid biopsy becomes part of the clinical practice [51]. One important limitation of liquid biopsy in oncology is the low specificity and sensitivity, since alterations are detected in body fluids, and not in the tumor itself [29].

### 2.2. Liquid Biopsy Biomarkers vs. Classical Biomarkers in Cancer Cachexia

Although liquid biopsy is commonly used and studied in cancer, it might be also applicable to several conditions, including cancer cachexia, to identify/diagnose it at an early stage. Liquid biopsy might be an alternative to muscle or adipose tissue biopsies to obtain information on the molecular changes occurring in these compartments over time and to monitor the process of skeletal muscle and adipose tissue atrophy in a less invasive way compared to surgical biopsies, especially in a research setting.

A prognostic indicator of the therapeutic response in cancer cachexia would be fundamental for the success of the treatment [21,22]. Considering that skeletal muscle represents the most affected organs in cancer cachexia and a main target for cachexia prevention and treatment, liquid biopsy biomarkers in cancer cachexia need to be, by definition, directly produced by skeletal muscle. Therefore, they would be much more specific with respect to classical cachexia biomarkers which are not necessarily produced by skeletal muscle.

It has been proposed that the mediators involved in the pathogenesis of CC, such as the pro-inflammatory cytokines IL-6 and TNF-α, hormones (e.g., leptin and ghrelin), and peptides, such as CAF [22,23], could be used as classical biomarkers in the development and progression of cachexia, although the exact role of these molecules has not been fully elucidated yet [23,25]. However, they are interesting because their concentration in the bloodstream of cachectic patients seems to be different compared to healthy subjects; e.g., ghrelin concentration decreases whereas pro-inflammatory cytokines, myostatin and growth and differentiation factor 15 (GDF-15) concentrations increase [52]. Due to the multifactorial nature of cachexia, a molecular mediator that fully satisfies the characteristics of an ideal biomarker:easily accessible, sensitive, reproducible and not expensivehas not yet been identified [21,22,23]. Among potential biomarkers of cancer cachexia, some are produced by skeletal muscle, which makes these molecules eligible as potential liquid biopsy biomarkers [23,51]. For example, CAF [53], collagen fragments, as well as the 3-methylhistidine derived from actin, myosin and titin fragments, released from atrophic muscles, might be considered as possible liquid biopsy biomarkers [54]. Similarly, myostatin (growth and differentiation factor 8; GDF-8), belonging to the transforming growth factor-β (TGF-β) superfamily, seems to play a critical role in cachexia being a negative regulator of muscle growth [23]. Since it is mainly produced by skeletal muscle, it might be a potential liquid biopsy biomarker [55] similar to some pro-inflammatory cytokines and some miRNAs also produced by this tissue.

## 3. miRNAs

Recent data [56,57,58] indicated that miRNAs and the alteration of the complex network between them and their mRNA targets could be responsible for the onset/progression of cachexia or for the systemic inflammation associated with this condition, although to date these points remain to be fully elucidated [6,7,24,40,51,59,60,61,62,63,64,65,66,67,68,69,70,71,72,73].

The miRNAs are included in the category of cfNAs, which were identified in human plasma for the first time in 1948 [29,59]. miRNAs are single-stranded, small non-coding RNAs with a size of ~22 nucleotides. They were discovered in 1993 and are present in all living organisms (viruses, algae, plants, invertebrates and vertebrates) [60,61,62].

In the present review we focused our attention on circulating, muscle-produced miRNAs detection through liquid biopsy as an alternative to invasive skeletal muscle solid biopsy. The reason for this is that miRNAs have many features in common with an ideal biomarker, among which they are stable in body fluids since they are resistant to RNAse digestion, to extreme pH, as well as to high temperatures and to repeated freeze-thaw cycles [32,41,74,75,76]. In addition, they are easily accessible through the repeatable and less invasive liquid biopsy method compared to the anatomical and clinical difficulties that can be encountered in performing a surgical biopsy [29,51].

### 3.1. Summary of the Biogenesis of miRNAs

**Figure 1:**
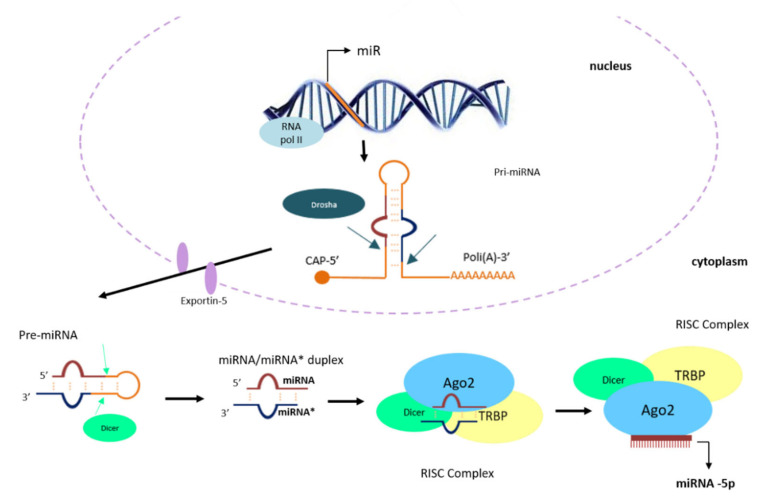
Biogenesis of microRNAs. Within the nucleus, RNA polymerase II synthesizes a long primary miRNA (pri-miRNA), from intronic, exonic or intragenic sequence. Pri-miRNA is characterized by a hairpin structure due to the pairing of inverted and close repetition. The microprocessor complex, consisting of Drosha (nuclear RNAse III) and Di George syndrome chromosomal region 8 (DGCR8), cleaves the pri-miRNA to form a precursor miRNA, a double helix called pre-miRNA. The pre-miRNA is exported to the cytoplasm in an Exportin-5/RanGTP-dependent manner. Dicer processes pre-miRNA into the cytoplasm by removing the terminal loop that forms the hairpin, giving rise to a miRNA/miRNA duplex *, consisting of a guide and passenger strand (indicated with an asterisk). Both strands originated from this miRNA maturation process are loaded onto ARGONAUTA2 (AGO2) protein in an ATP-dependent manner, to form an effector complex called RNA-Induced Silencing Complex (RISC). The RISC Complex is formed by the miRNA/miRNA * duplex loaded to the AGO2 protein, but also by Dicer and the TAR RNA-binding protein (TRBP) protein, which contribute to the formation of the mature RISC complex. In the formation of the effective, mature RISC complex, AGO2 unwinds and cleaves the passenger strand, giving rise to a mature single-stranded miRNA, which will perform the regulatory function. Two mature miRNAs could be originated from the pri-miRNA; one from the 5′ arm and one from the 3’ arm of the hairpin thus they take the suffix -5′ or -3′ respectively.

The biogenesis of miRNA begins within the nucleus where the RNA polymerase II synthesizes a primary transcript, called pri-miRNA which undergoes several maturation processes. Pri-miRNAs have a length of about 100 nucleotides and can be transcribed by their promoters, located in intragenic (mostly introns but also exons) and intergenic regions [77,78]. Pri-miRNAs are characterized by inverted and close repetitions that pair forming a hairpin structure (Figure 1).

Two mature miRNAs could originate from the pri-miRNA; one from the 5′ arm and one from the 3’ arm of the hairpin, therefore they take the suffix -5′ or -3′, respectively [79] (Figure 1). In some cases, a single, long primary transcript gives rise to more than one mature miRNA; they form a cluster and the miRNAs contained in it constitute a family. For example, miR-29a, miR-29b and miR-29c belong to the miR29 family [80,81,82].

Pri-miRNAs are cleaved by a microprocessor multiprotein complex consisting of Drosha, a nuclear RNAse III, and the double-stranded RNA binding protein Pasha/Di George syndrome chromosomal region 8 (DGCR8) [83], which allows the formation of a short double helix called pre-miRNA [79] which migrates from the nucleus to the cytoplasm via exportin-5 (Figure 1). Into the cytoplasm, the RNAse III Dicer removes the hairpin that joins the 3′ and 5′ arm, producing a mature miRNA/miRNA * duplex, consisting of a guide and of a passenger strand (here indicated with an asterisk). Some RNA-binding proteins, including the endoribonuclease Argonauta2 (AGO2), bind the miRNA duplex to form the RNA-Induced Silencing Complex (RISC), which unwinds the duplex of miRNA, leaving the guide strand bound to the AGO2 protein ready to perform its regulatory function, while the passenger strand is degraded [84] (Figure 1) The selection of which strand of the miRNA/miRNA * duplex will be the guide and which one the passenger strand, is thought to be determined by their thermodynamic stability and by their composition. Generally, the guide strand is the one with the lower thermodynamic stability at the 5’ end or the one containing an uracil at the 5’ end [85,86,87]. The guide strand is usually the more prevalent and more biologically active among the two [79].

As recently reported by O’Brien and colleagues [84], a “non-canonical” miRNA biogenesis pathway has also been identified. In this pathway, miRNA’s processing is independent from Drosha/DGCR8 and/or Dicer. Nevertheless, the final result is the same; the protein AGO2 binds the mature miRNA to form the RISC complex [84].

In the RISC complex, the single stranded miRNA associates with its target mRNA having a complementary sequence. By this miRNA-mediated mechanism, the regulation of gene expression occurs at the post-transcriptional level, through the degradation or the inhibition of the target mRNA translation [88,89]. A miRNA can silence many mRNAs, and a single mRNA can be the target of multiple miRNAs. Generally, the miRNA binds the 3’UTR region of the mRNA, however, although less frequently, miRNA interactions with the 5’UTR region and coding regions have also been reported [90]. The silencing mechanism induced by the miRNA depends on the degree of complementarity between the miRNA and target mRNA: a decreased complementarity determines the inhibition of the translation, whereas a perfect complementarity causes the degradation of the mRNA [91]. It has been shown that miRNAs are also capable of binding promoter regions, thus inducing gene transcription [92]; however, more studies are required to better understand the underlying mechanism.

### 3.2. Circulating miRNAs and Their Biological Implications

**Figure 2:**
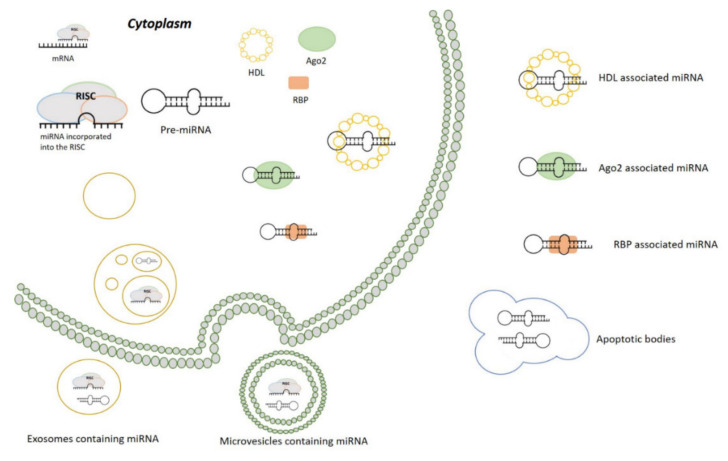
microRNA release mechanisms. miRNAs can be released into the extracellular space through several mechanisms: both pre-miRNAs and mature miRNAs, loaded into the RISC complex, can be incorporated into exosomes or microvesicles and secreted from the donor cell. Circulating miRNAs can also be derived from apoptotic bodies. Moreover, miRNA can be stable in biological fluids in association with RNA-binding proteins (RBP), Ago2 or HDL.

miRNAs were first identified in tissues and subsequently in liquid biopsies. The total concentration and the composition of miRNAs vary among the different biological fluids. Identification of extracellular miRNAs generated high interest in the scientific community because biological fluids contain RNAses, enzymes that perform a protective function against exogenous nucleic acids [37]. It has been proposed that miRNAs are very stable in biological fluids because they can escape from the RNAses activity, through two possible ways: being packaged in extracellular vesicles (EVs) or being associated with proteins [93] (Figure 2). In vitro studies have shown that muscle-derived miRNAs can be released into the bloodstream [6,7,73], making these molecules potential indicators of muscle metabolism.

#### 3.2.1. miRNAs Packaged in EVs

EVs are small particles surrounded by a plasma membrane and containing several cytosolic molecules (miRNA, mRNA, DNA, proteins and lipids) derived from the donor cells [31,94]. RNAses cannot enter these vesicles [40].

EVs constitute a heterogeneous population of particles of different sizes, biochemical composition, density, tissue of origin, function and biogenesis [95,96]. Recently, EVs have been classified into three classes named exosomes, ectosomes/microvesicles and apoptotic bodies, based on their size and mechanism of biogenesis [97].

Exosomes are nano-sized vesicles (30–150 nm) of endosomal origin, which are released in the extracellular environment by fusion between endocytic vesicles and plasma membrane through an active and finely regulated process. They originate by the fusion between an early endosome, also called multivesicular body (MVB), and plasma membrane [40,97,98]. All cell types are able to release exosomes, and the exosome cargo allows to identify the originating cell [40,99].

Ectosomes (also called microvesicles) (100–1000 nm) originate from the outward budding of the plasma membrane [100]. Following an extracellular signal, the donor cell forms a protrusion of the plasma membrane whose detachment originates these vesicles [101,102].

Apoptotic bodies are vesicles larger than 800 nm, produced by fragmentation of the apoptotic cell which releases these vesicles into the extracellular space [103,104]. Some of EVs are the oncosomes, vesicles containing miRNA, mRNA and proteins that are actively produced by cancer cells and released into biological fluids [95,99,105,106]. Once released into body fluids, miRNAs can act as messengers and regulate cell functions in an autocrine, paracrine or endocrine manner [9,95,102,107,108,109]. Interestingly, experimental studies showed that miRNAs contained in EVs can trigger muscle atrophy in vitro [7,73].

#### 3.2.2. miRNAs Associated with Protein

The second way in which miRNAs are able to escape from the activity of RNAses is through their association with proteins. Arroyo and colleagues [33] were the first to demonstrate that miRNAs are released into biological fluids not only packaged in vesicles. They speculated that miRNAs are associated with elements that made them stable in the blood, protected against the RNAses activity [33]. Subsequently, they revealed the presence of AGO2 in body fluids, demonstrating that miRNAs can be associated with ribonucleoprotein complexes giving them stability. Other studies showed that miRNAs in body fluids can also be bound to the protein nucleophosmin, as well as to lipoproteins (HDL and LDL) [33,109,110,111,112,113,114,115,116,117].

Nowadays we still do not know the exact mechanisms of miRNA’s export systems from cells. Two possible mechanisms have been hypothesized, a passive and an active one [111]. The passive mechanism allows the release of miRNAs as by-products of cells, both packaged in EVs or associated with proteins [97,118], for example following apoptosis or necrosis [32,74,103,119,120,121]. On the other hand, miRNAs can be released as EVs and EVs-free forms, by an active mechanism [33,112,120,122,123]. Noteworthy, in vitro studies showed that miRNAs can also be exchanged between cells through gap junction or through direct cell-to-cell contacts [117].

These mechanisms represent a strategy of cell-to-cell communication and gene expression regulation between cells located at a considerable distance in the organism [120,121,124,125]. In this light, analyzing the levels of muscle-derived miRNAs released in biological fluids (both EVs and EVs-free form), could allow us to obtain information on muscle metabolism during cancer cachexia.

## 4. Skeletal Muscle-Derived miRNAs as Potential Candidate for Liquid Biopsy Biomarkers in Cancer Cachexia

### 4.1. Murine and Human Circulating Muscle-Derived miRNAs as Liquid Biopsy Biomarkers for Cancer Cachexia

The loss of muscle mass during cachexia is determined by an imbalance between the rates of protein synthesis and degradation, in addition to several metabolic changes occurring systemically in the muscle [25,70,71,126,127,128,129,130].

Many researchers have focused on the role of muscle-derived miRNAs in muscle growth and muscle atrophy pathways, as also reported by Donzelli et al. [131]. miRNAs expressed in skeletal muscle such as miR1, miR133a, miR133b, miR206 and miR486 were found to be involved in cancer cachexia. They directly modulate the processes of myogenesis, growth, differentiation and apoptosis of myotubes, as well as protein synthesis and skeletal muscle homeostasis [132,133,134,135,136,137]. Myo-miRNAs are specifically expressed in skeletal muscle since their genes are either located into the intronic regions of the Myosin Heavy Chain (MyHC) genes or they are directly controlled by specific muscle transcription factors such as Myoblast Determination protein 1 (MyoD), Myocyte enhancer factor-2 (Mef2), or Serum responsive factor (Srf) [138,139,140,141,142].

**Figure 3:**
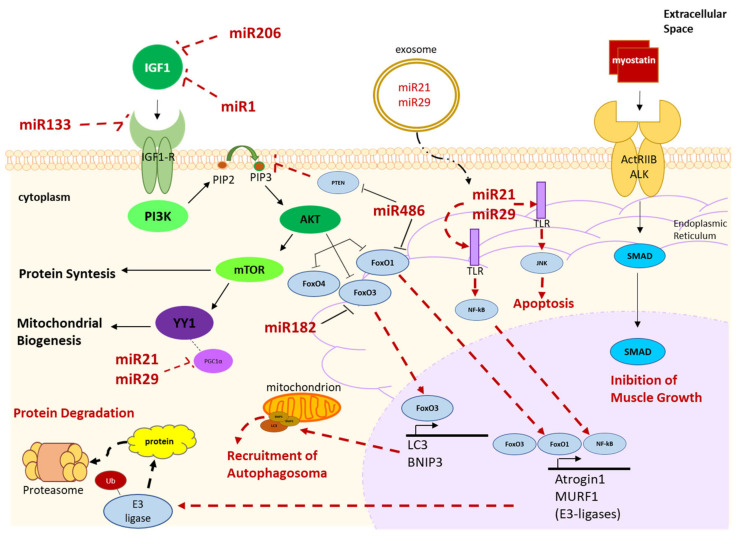
Schematic representation of miRNAs involved in the regulation of atrophy and protein degradation pathways in skeletal muscle tissue. Insulin-like growth factor 1 (IGF-1) activates the Akt/mTOR signaling pathway, responsible for protein synthesis. Akt inhibits the degradation of proteins by keeping inactive in the cytoplasm transcription factors members of the forkhead box protein O (FoxO) family (FoxO1, FoxO3 and FoxO4), which otherwise migrate in the nucleus and promote the transcription of the E3 ligases (Atrogin1 and MuRF1), responsible for the protein degradation mediated by the system of the ubiquitin proteasome. In the nucleus FoxO3 is also able to promote the transcription of microtubule-associated protein 1A/1B-light chain 3 (LC3) and BCL2 Interacting Protein 3 (BNIP3) which once in the cytoplasm localize on the outer membrane of the mitochondria, promoting mitophagy through the recruitment of the autophagosome. The Akt/mTOR pathway is also able to promote the activation of the factor Yin Yang 1 (YY1) which, associating with peroxisome proliferator activated receptor gamma coactivator 1 alpha (PGC1α), induces mitochondrial biogenesis. Akt activation is inhibited by phosphatase and tensin homolog (PTEN) which inhibits the phosphorylation of PIP2 to PIP3. Several in vitro and in vivo studies have shown that elevated circulating levels of miR-1, miR-133a/b and miR-206 appear to negatively regulate the IGF-1/Akt/mTOR pathway, by targeting positive regulators IGF-1 and IGF1-receptor (IGF-1R) thus promoting muscle wasting. On the contrary, miR-486 and miR-182 promote protein synthesis, by targeting negative regulators of this signaling pathway (PTEN, FoxO1-3). miR-21 and miR-29 contained in the exosomes can penetrate the muscle fiber and activating the TLR7/8 located on the endoplasmic reticulum promoting i) the activation of JNK which induces apoptosis of the muscle fiber and ii) NF-kB which, migrating in the nucleus, promotes the transcription of Atrogin1 and MuRF1, responsible for protein degradation. miR21 and miR29 in skeletal muscle tissue are also able to negatively regulate mitochondrial biogenesis by targeting YY1. Finally, the signaling pathway induced by myostatin activates SMADs (2-3-4) which, by moving to the nucleus, inhibit muscle growth. Dashed arrows indicate the pathways that lead to muscle wasting. Dashed lines indicate the inhibitory function of miRNAs in skeletal muscle tissue. Black arrows indicate the pathways involved in muscle homeostasis.

As will be specifically described below, the circulating levels of these muscle-expressed miRNAs are modulated during cancer cachexia [2,3,4,5,6,7,71,143]. For this reason, they are gaining increasing interest in liquid biopsy in cancer cachexia [144,145,146,147]. (Figure 3).

#### Murine and Human Circulating Muscular miRNAs Involved in the Insulin-Like Growth Factor 1 (IGF1)/Akt/mTOR Pathway

*miR1*, *miR133a*/*b and miR206* are skeletal muscle-expressed miRNAs since they are all expressed in skeletal muscles [148,149]. miR1 and miR133a are also produced by the cardiac muscle [150,151]. In vitro experiments on both *human* and *mouse* muscle cells showed that high levels of miR1 and miR133a/b are released by myotubes in extracellular space [6,7,148,149]. Interestingly, as discussed below, the levels of these miRNA are modulated in mouse models of muscle wasting conditions both in the skeletal muscle tissue, thus suggesting an autocrine/paracrine function, and in the blood thus suggesting an endocrine role for them [6,7,71,72,143].

The miR1-1/miR133a-2, miR1-2/miR133a-1 and miR133b/miR206 [132] are clustered in different genes/chromosomes. However, although two miRNAs are in the same cluster, their transcription can take place separately [152]. miR1 is mainly expressed in the fast-twitch glycolytic fibers while miR206 is expressed in the slow-twitch oxidative fibers [153,154]. Both miRNAs have the same molecular target, IGF1 (Figure 3). Therefore, high circulating levels of miR1 and miR206 block the IGF1/Akt/mTOR pathway, which is the main pathway regulating myofiber size by activating protein synthesis and mitochondrial biogenesis [155,156] (Figure 3). Blocking the IGF1/Akt/mTOR pathway results in increased protein degradation and muscle atrophy [148,149,157]. The same signaling pathway is inhibited by high circulating levels of miR133a/b since its molecular target is the IGF1 receptor (IGF1-R) [148,149] (Figure 3).

Accordingly, Cacchiarelli and colleagues observed elevated levels of miR1, miR133 and miR206 in the blood of patients with Duchenne muscular dystrophy, whereas the expression levels of these miRNAs in skeletal muscle tissue of the same patients were low [158]. Moreover, Köberle [159] hypothesizes that the progression of cachexia could lead to a reduction in circulating myo-miRNAs (especially miR1), due to advanced muscle wasting [147]. Interestingly, miR1 and miR206 have also been suggested to be involved in myogenic differentiation. In fact, MyoD and Myogenin seem to promote the over-expression of miR1 and miR206 and high levels of these miRNAs have been found to decrease the expression of Pax7 compared to MyoD, thus resulting in increased satellite cell differentiation [127,129,140,160,161,162].

The *miR486* is defined “muscle-enriched” because its expression level is higher- but not exclusive- in skeletal muscle relative to other tissues [163]. It has autocrine/paracrine effects; in fact, in physiological conditions, miR486, in myofibers, keeps forkhead box protein O 1 (FoxO1) inactive, thus dampening autophagy and muscle atrophy [164] (Figure 3). Another molecular target of miR486 is represented by phosphatase and tensin homolog (PTEN) which acts by inhibiting the phosphorylation of PIP2 to PIP3, thus blocking the activation of the AKT/mTOR pathway (Figure 3). Under normal physiological conditions miR486 decreases the expression of PTEN, thus allowing protein synthesis [165]. Therefore, the down-regulation of this miRNA at tissue level leads to a decrease in protein synthesis and to an increased expression of Atrogin1 and MuRF1 mediated by FoxO1, which leads to myofiber atrophy [148,165] (Figure 3). The expression of miR486 seems to be regulated by MyoD and its over-expression would appear to promote the differentiation of myoblasts [145,146]. Xu and colleagues (2012) demonstrated that high expression of miR486 in skeletal muscle tissue improve muscle mass of mice models of chronic kidney disease [6,164]. This muscle-enriched miRNA has also been found to be circulating, both in humans and mice [6,164,166,167].

### 4.2. Other Human Skeletal miRNAs as Potential Future Liquid Biopsy Biomarkers for Cancer Cachexia

Other skeletal muscle-derived miRNAs have been more recently found to be modulated in cancer cachexia, also having in most cases an important prognostic value [131,168,169].

However, in order to be used as liquid biopsy biomarkers, it will be necessary to assess their presence also in body fluids during cachexia. It will also be important to evaluate if their circulating moieties are modulated during cancer cachexia, like their skeletal muscle counterpart do and also if they derive from skeletal muscle; this issue might be investigated analyzing the miRNAs contained into EVs, since the origin of EVs might be confirmed by analyzing their surface markers [97]. Once established that a specific modulated circulating miRNA is released by the skeletal muscle, this could be used as liquid biopsy biomarker and directly amplified on the total RNA extracted by the liquid biopsy. On the other hand, if a specific miRNA is released by several different tissues, then EVs isolation and identification, followed by miRNA quantification should be performed. However, it has to be taken into account that exosomes were observed to contain a small minority of the miRNA content in plasma. Therefore, it has been suggested that exosomes are individually unlikely to be vehicles for miRNA-based intercellular communication [170]. In this perspective, we acknowledge that this is a complex procedure difficult to be implemented routinely as a prognostic tool.

In 2017, a study conducted on human rectus abdominus biopsies identified eight tissue-related miRNAs (*let-7d-3p*, *miR199a-3p*, *miR345-5p*, *miR423-5p*, *miR423-3p*, *miR532-5p*, *miR1296-5p* and *miR3184-3p*) upregulated in cachectic cancer patients compared to non-cachectic cancer patients [144]. If this difference was also found in the bloodstream, these miRNAs might be considered potential biomarkers of muscle wasting during cancer cachexia.

More recently, other skeletal muscle miRNAs (*miR18a*, *miR208a*, *miR208b*, *miR422a*, *miR499*, *miR542-5p* and *miR542-3p*) have been proposed to play a role in the pathophysiology of cancer cachexia [131,168]. In particular, both in vitro and in vivo studies (*human* samples but also *mouse models*) showed overexpression of *miR18a*, *miR422a*; *miR542-5p*/*3p* in skeletal muscle tissue during muscle wasting conditions [168]. However, in silico analysis [131] and other in vivo experiments on mice [171] showed that decreased expression levels of *miR208a* and *miR499* in skeletal muscles were related to decreased muscle mass [131,171]. Furthermore, van de Worp et al. [169] found additional miRNAs modulated in vastus lateralis muscle of lung cancer patients with cachexia (*miR-424*/*miR-503* cluster and the *miR144-3p*/*451a* cluster). The first miRNA cluster includes *miR424-5p*, *miR424-3p*, *miR450a-1*, *miR450a-2*, *miR450b*, *miR503*, *miR542-5p*, *miR542-3p* [169]. They reported an overexpression of miR424-3p, miR424-5p and miR450a, whereas miR144-5p and miR451a were downregulated in cachectic cancer patients [169]. Based on the results obtained both in silico analysis and in experimental models (in vitro and in vivo, *humans*), the downregulation of miRNAs *miR27a*/*b* and *miR182* seems to be associated with cancer cachexia [81,143,149,168].

#### 4.2.1. Human Muscle-Derived miRNAs Involved in the IGF1/Akt/mTOR Pathway

*miR18a*, *miR199a* and *miR345-5p* regulate the IGF1/Akt/mTOR pathway and induce the expression of MuRF1 and Atrogin1, thus leading to a reduction in protein synthesis and to muscle atrophy [64,144,168,172,173,174,175,176,177]. They have therefore an autocrine/paracrine role. Besides the IGF1/Akt/mTOR pathway, another molecular target of miR345-5p is the extracellular matrix (ECM) protein, alpha-1 type I collagen (COL1A1), whose down-regulation in skeletal muscles of cachectic patients, is associated with muscle atrophy [144]. Furthermore, another target of miR345-5p is the Transferrin Receptor (TFRC), which was down-regulated in the muscle of these patients [144]. Transferrin influences skeletal muscle homeostasis, and it has been speculated that its down-regulation might lead to an imbalanced myogenic differentiation [144,178]. Similarly, miR486, miR182 has FoxO3 as its molecular target and regulates the process of autophagy and atrophy in skeletal muscle [143].

#### 4.2.2. Human Muscle-Derived miRNAs Involved in Myogenic Differentiation

The *Let-7d-3p* belongs to a miRNA family called Let-7. Similarly, to miR345-5p, Let-7d-3p targets TFRC, that might affect myogenic differentiation which leads to skeletal muscle wasting [144]. The *miR1296* is involved in the serotonin-mediated signaling pathway regulating myogenic differentiation [179]. Moreover, miR1296-mediated modulation of serotonin seems to be linked to food intake reduction [180].Also, *miR3184-3p* influences myogenic differentiation by regulating the WNT/β-catenin signaling pathway [144].

#### 4.2.3. Human Muscle-Derived miRNAs Involved in the Myostatin/Smad Pathway

Based on in silico predictions, the target of *miR208a*/*b*, *miR422a miR499* and *miR542-5p*/*3p* might belong to the Myostatin/Smad pathway, a major pathway negatively regulating muscle growth [131,149,168,181]. *miR27a*/*b* also seems to be involved in the Myostatin signaling pathways although there are few experimental data confirming this function [148,149,168].

#### 4.2.4. Human Muscle-Derived miRNAs Involved in Skeletal Muscle Homeostasis

The *miR423-5p* and *miR532-5p* are regulators of the energy metabolism [144]. In fact, miR423-5p modulates the intramuscular levels of leptin, while a molecular target of miR532-5p is the Neuro Peptide Y Receptor (NPYR), whose down-regulation could contribute to the pathophysiology of cancer cachexia [144]. *miRNA424-5p* seems to have as molecular targets proteins involved in the rRNA synthesis, thus modulating the translation machinery [168]. However, although modulations of miRNAs belonging to these clusters are reported in different types of cancer, their function is not yet fully understood.

The results reported in these studies indicate an autocrine/paracrine function for all the human miRNAs described in this section; experimental studies confirming the presence of circulating levels of such miRNAs are needed to propose an endocrine function for them and their potential utility as liquid biopsy biomarkers for cancer cachexia.

### 4.3. Other Skeletal miRNAs Identified in Mouse Models of Cancer Cachexia

Finally, data indicate the involvement of other miRNAs modulated in skeletal muscle of cancer cachexia mice models such as *miR223-3p, miR229a-3p miR299a-3p, miR431-5p, miR511-3p*, *miR665-3p*, *miR1933-3p* and *miR3473d*, as well as *miR147-3p* and *miR205-5p* [71,182]. Specifically, Lee et al. found the upregulation of miR147-3p, miR511-3p, miR223-3p and miR205-5p in the tibialis anterior of cancer cachexia mouse models, whereas other six miRNAs were downregulated in these muscle samples [71]. This suggests an autocrine/paracrine function for these miRNAs in this setting.

Most of these skeletal muscle-derived miRNAs have also been detected in the bloodstream [40,183,184,185,186,187,188,189,190,191,192,193,194,195,196,197,198,199]. However, to date it is unknown whether their circulating levels are modulated during cancer cachexia in humans. Further studies are necessary to clarify this point which appears crucial in order to evaluate the potential role of these miRNAs in liquid biopsy for cancer cachexia.

## 5. From Research to Clinical Practice: Steps to Be Improved in Order to Use Circulating Skeletal Muscle Derived-miRNAs in Liquid Biopsy

In physiological conditions, muscular and circulating muscular miRNAs maintain skeletal muscle homeostasis and are expressed at low concentrations in biological fluids [163], while their up- or down-regulation has been observed in several diseases [41,200,201,202,203]. While promising results have been achieved by using experimental cachexia models, results on miRNAs obtained in humans are controversial and need more effort to become definitive due mainly to the higher variability of cachectic patients. Robust data are necessary to standardize a protocol to allow the greatest recovery of miRNA and minimizing hemolysis in serum or plasma to use liquid biopsies as a source of circulating skeletal muscle derived-miRNAs [204,205,206]. To date, the choice of serum or plasma as the better source for circulating miRNA extraction is still debated. According to Vigneron and colleagues, the presence of EDTA in the plasma tube could inhibit the precipitation of RNA and reduce hemolysis [207]. To note, miRNA16-5p is very often used as an endogenous control and differences in the concentration of this miRNA in serum and plasma make non-univocal the interpretation of the results [207].

Noteworthy, in addition to the variability associated to the different types of biological fluids used as liquid biopsy, there is also a variability between the methods and kits used to perform the analysis [31,207,208,209] that may influence the yield and quality of the extracted RNA [149], thus indicating discrepancies related to the method used rather than a real difference [207,208,209]. To date, quantitative Real-Time PCR is the “gold standard” for miRNA analysis [147], however, there are several kits (also very different from each other) available to perform retro-transcription (RT) and Real-Time PCR steps. Standardized methods for collecting biological fluids, for RNA extraction and for the RNA quantification would make possible the comparison of data obtained by different researchers [41,51,207,210].

Importantly, in order to use circulating miRNAs in liquid biopsy, it is necessary to identify a reliable endogenous control that is constitutively and stably expressed in all subjects and that is not modulated during pathological conditions [146]. Some research groups have recently used a combination of two or more miRNAs as endogenous controls [211,212,213]. Moreover, several circulating miRNA extraction kits provide the use of an exogenous control [211,214], a non-human miRNA, with a known concentration, to be added in the miRNA extraction phase. Comparing the data relative to the exogenous control with those relative to the endogenous control, it would be possible to verify the efficacy of both the chosen endogenous control and the execution of the method [130,201,211,214,215].Also, it appears necessary to identify the reference values for circulating miRNAs in healthy subjects in order to clarify the variability related to different factors including sex, ethnicity, age, diet, physical activity and all the factors influencing the expression of circulating miRNAs [146,147].

## 6. Conclusions

The diagnosis of cancer cachexia occurs generally late if compared to the onset of the profound metabolic alterations leading to loss of muscle mass and to reduced food intake typical of this syndrome. For this reason, it is crucial to identify reliable biomarkers to be detected in a non-invasive manner and allowing an early diagnosis of cachexia. In this light, it could be possible to develop targeted interventions to improve the outcomes. The discovery of circulating miRNAs and the use of liquid biopsy has opened a new, interesting scenario in the field of cancer research. Circulating miRNAs could play a key role as biomarkers in revealing the onset and progression of cachexia, which is characterized by systemic metabolic and nutritional alterations. Several studies on animal models showed an involvement of muscular miRNAs in the pathophysiology of cancer cachexia. However, there are discrepancies among the studies on the role of miRNAs in human cachexia. These limits are due both to the high variability of cachectic cancer patients and to the lack of standardized protocols for miRNA’s analysis. Furthermore, the mechanisms identified in experimental animal models might not always exactly reflect the molecular interactions occurring in humans.

For this reason, increasing the knowledge about the miRNA pathways and tissue/organs cross-talk, may allow the application of miRNAs as diagnostic and prognostic biomarkers in the setting of cancer cachexia.

## Figures and Tables

**Table 1:** Advantages and disadvantages of liquid biopsy.

	Advantages	Disadvantages
	Needs a small volume of blood (usually 6–10 mL)	Techniques not yet standardized
	Not invasive	Clinical practice rules are not yet established
	Easily repeated	It is not reliable for all types of cancer (e.g., lung cancers), the diagnosis and subtyping can be established by only histology
LIQUID BIOPSY	Useful to reveal spatial and temporal tumor heterogeneity	May not be representative of the whole cancer
	Real time monitoring for drug response and resistance Timely search for genotyping mutations	

## Data Availability

No new data were created in this study.
